# Insights into the Antimicrobial Mechanism of Action of Human RNase6: Structural Determinants for Bacterial Cell Agglutination and Membrane Permeation

**DOI:** 10.3390/ijms17040552

**Published:** 2016-04-13

**Authors:** David Pulido, Javier Arranz-Trullén, Guillem Prats-Ejarque, Diego Velázquez, Marc Torrent, Mohammed Moussaoui, Ester Boix

**Affiliations:** Department of Biochemistry and Molecular Biology, Faculty of Biosciences, Universitat Autònoma de Barcelona, E-08193 Cerdanyola del Vallès, Spain; javier.arranz@e-campus.uab.cat (J.A.-T.); guillem.prats.ejarque@uab.cat (G.P.-E.); diego.velazquez@uab.cat (D.V.); marc.torrent@vhir.org (M.T.); mohammed.moussaoui@uab.cat (M.M.)

**Keywords:** RNases, host defence, antimicrobial peptides, cell agglutination, infectious diseases

## Abstract

Human Ribonuclease 6 is a secreted protein belonging to the ribonuclease A (RNaseA) superfamily, a vertebrate specific family suggested to arise with an ancestral host defense role. Tissue distribution analysis revealed its expression in innate cell types, showing abundance in monocytes and neutrophils. Recent evidence of induction of the protein expression by bacterial infection suggested an antipathogen function *in vivo*. In our laboratory, the antimicrobial properties of the protein have been evaluated against Gram-negative and Gram-positive species and its mechanism of action was characterized using a membrane model. Interestingly, our results indicate that RNase6, as previously reported for RNase3, is able to specifically agglutinate Gram-negative bacteria as a main trait of its antimicrobial activity. Moreover, a side by side comparative analysis with the RN6(1–45) derived peptide highlights that the antimicrobial activity is mostly retained at the protein N-terminus. Further work by site directed mutagenesis and structural analysis has identified two residues involved in the protein antimicrobial action (Trp1 and Ile13) that are essential for the cell agglutination properties. This is the first structure-functional characterization of RNase6 antimicrobial properties, supporting its contribution to the infection focus clearance.

## 1. Introduction

**Figure 1:**
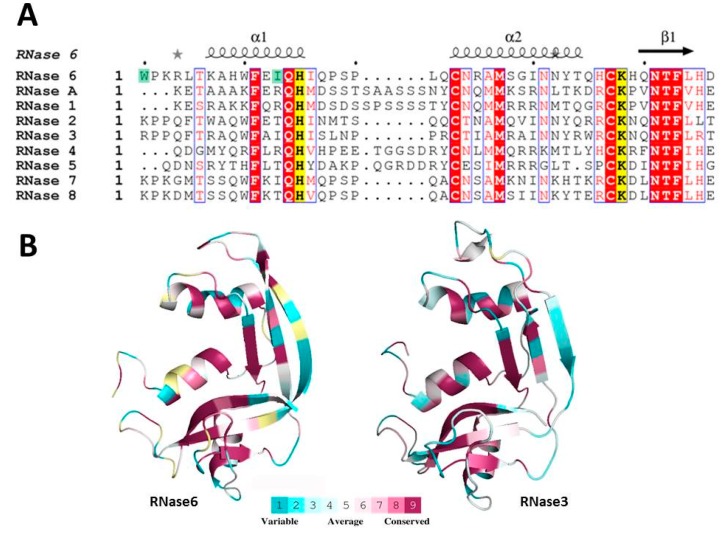
(**A**) Structure-based sequence of the eight canonical human RNases N-terminal domain together with RNaseA. The active site residues are highlighted in yellow. RNase6 tested mutations related to antimicrobial activity are labeled in green. The alignment was performed using *ClustalW*, and the picture was drawn using *ESPript* [33]. Labels are as follows: red box, white character for strict identity; red character for similarity in a group and character with blue frame for similarity across groups (red box, white character: strict identity; red character: similarity in a group; red character with blue frame: similarity across groups; yellow box, black character: catalyst residue); (**B**) RNase6 (PDB 4X09; [34]) and RNase3 (PDB 4A2O; [35]) three-dimensional structure surface representations using the CONSURF web server ([36]) featuring the relationships among the evolutionary conservation of amino acid positions in the RNaseA family. The three-dimensional structure shows residues colored by their conservation score using the color-coding bar at the image bottom.

The RNaseA superfamily is a vertebrate-specific gene family that comprises a wide set of secreted ribonucleases displaying a variety of biological properties [1,2]. In particular, distant related members were reported to share innate immunity properties, suggesting that the vertebrate RNases have evolved as a host-defense family [3,4,5]. Eight functional members are found in humans, known as the “canonical RNases” (Figure 1), sharing a common structural fold and catalytic triad [6]. Within the family, we can differentiate three main phylogenetic lineages (RNase5, RNases2/3 and RNases6–8) related to host defense [7,8,9,10]. The eosinophil ribonucleases, EDN (eosinophil derived neurotoxin, RNase2) and ECP (eosinophil cationic protein, RNase3), are two secretory ribonucleases stored in the secondary granules of eosinophils and released at the focus of infection [11,12]. *RNase2* and *3* genes have diverged after gene duplication, accumulating rapidly non-silent mutations through positive selection pressure [13,14]. RNase2 acts as a potent modulator of the immune host system, and additionally displays a high antiviral activity against rhinovirus, adenovirus and syncytial respiratory virus in a RNase catalytic activity dependent manner [15,16,17]. RNase3 possesses a highly antimicrobial activity against bacteria [11,18,19,20,21], and many parasites, such as helminths and protozoa [22]. By contrast, the antimicrobial properties of RNase3 are not dependent on the ribonuclease activity of the protein [18,23]. On the other hand, RNase7 is another RNase secreted by a variety of epithelial tissues [24] and displaying a high antimicrobial activity against a wide range of bacteria, regarded as a major contributor to the skin barrier protection [25,26,27,28]. In turn, RNase6 has been related with the host immune system protection, being expressed in neutrophils and monocytes and displaying a high antimicrobial activity [29]. Recently, it has been reported that RNase6 and RNase7 play an important role in bacterial clearance at the urinary tract [30]. Nonetheless, our understanding of the antimicrobial mechanism of action of the RNase6 is still poor.

Although the secreted vertebrate RNases share an overall globular three-dimensional prototypical scaffold, a catalytic triad and a particular disulfide pattern, their amino acid sequence identity ranges from 30% to 70%. Notwithstanding, despite the low sequence conservation among some RNases homologues, conserved structural features at the N-terminal region correlated to their host-defense properties [3]. Comprehensively, some human RNases are endowed with the features present on antimicrobial proteins and peptides (AMPs), sharing a marked cationicity that facilitates the electrostatic interaction with the negatively charged bacterial surfaces, abundance of hydrophobic residues, and the presence of dynamic amphipathic modules that can adopt secondary structures upon interaction with the bacterial envelopes [7,31]. Amidst the antimicrobial human RNases, despite their importance in the innate immune system, some members are poorly characterized and their antimicrobial features are yet to be described [17,28,30,32]. That is the case of the human RNase6, which is a small cationic protein mainly expressed in neutrophils and monocytes [29]. Interestingly, human RNase6 has been recently described as a key player in the protection of the urinary tract [30]. In spite of these encouraging findings, little is known about the antimicrobial mechanism of action of this RNase during infection.

In this work, we have characterized the antimicrobial mechanism of action of the human RNase 6 at both cell wall and membrane levels by describing its bactericidal effect against Gram-positive and -negative bacteria using a wide range of biophysical and microscopy approaches. Our results highlight that the antimicrobial properties of the protein are comparable to its RNase3 homolog and correlate to the bacterial cell damage and agglutination activities. Additionally, the bactericidal membrane leakage and bacterial agglutination properties of the protein are largely retained at its N-terminal domain.

## 2. Results

In order to evaluate the antimicrobial mechanism of action of RNase6, we used different experimental approaches that combined the analysis of the protein activity in synthetic lipid bilayers with its action on bacteria cultures. The antimicrobial properties of the protein and its N-terminus derived peptide were evaluated against Gram-positive and Gram-negative species. Additionally, we have also evaluated the protein affinity for bacterial cell wall lipopolysaccharides (LPS). Finally, N-terminus mutant variants were designed and their bactericidal activity and cell agglutinating properties were compared to the wild-type protein. All the results have been compared to the previously characterized human RNase3, taken as a positive control [21]. On the other hand, the family reference protein (RNaseA) did not display any of the tested antimicrobial and membrane damage activities at the same assayed conditions [37,38,39,40].

### 2.1. Membrane and Cell Wall Interaction

Our first approach to define the antimicrobial mechanism of action of the human RNase6 and its derived N-terminal peptide RN6(1–45) was performed by the characterization of the interaction at the membrane and cell wall levels.

By monitoring the intrinsic tryptophan fluorescence signal of the proteins and peptide upon incubation with phospholipid vesicles and LPS micelles, we were able to record the blue-shift in the tryptophan spectra, that this residue experiences when is embedded in a hydrophobic microenvironment. Thus, in order to assess the protein ability to interact with phospholipid bilayers we registered the intrinsic tryptophan fluorescence signal and the λmax shift in presence of charged (1,2-dioleoyl-sn-glycero-3-phosphoglycerol (DOPG)), neutral (1,2-dioleoyl-sn-glycero-3-phosphocholine (DOPC)) and mixed (DOPC/DOPG) liposomes (Table 1). The recorded spectrum for both RNases and the RNase6 derived N-terminal peptide showed no significant blue-shift upon incubation with non-charged large unilamellar vesicles (LUV). On the other hand, significant *λ*max shift towards the blue was experienced when incubated with both charged and mixed LUV. In addition, the two proteins and the assayed peptide also underwent a blue-shift in their emission spectra in the presence of LPS micelles, thereby indicating their ability to interact with the negatively charged bacterial cell membrane and envelope components.

To further characterize the interaction of RNase3, RNase6 and RN6(1–45) with the bacterial cell wall we performed a fluorimetric assay using the BODIPY^®^ cadaverine (BC) probe. Lipopolysaccharide binding affinity was determined as a result of the competitive displacement of BC, which mimics the lipid A portion of the LPS. The binding affinities of RNase3, RNase6 and its N-terminal derived peptide RN6(1–45) for the LPS molecule as a function of the protein concentration were tested. Their 50% effective dose (ED_50_) values, defined as the concentration for which half BC displacement occurs, and the total BC displacement (shown as a percentage) are displayed in Table 2. Both RNases and the derived peptide displayed a high affinity with the negatively charged LPS molecule, being able to totally displace the BC molecule at a micromolar range.

After defining the interaction at the cell wall and membrane levels; we further characterized the proteins and peptide ability of causing membrane disruption and cell agglutination. Therefore, LUV containing the fluorescent probe 8-aminonaphthalene-1,3,6-trisulfonic acid/p-xylenebispyridinium bromide (ANTS/DPX) were incubated with RNase3, RNase6 and RN6(1–45) and membrane disruption was recorded as a function of the fluorescence increment. Both human RNases were able to totally disrupt the ANTS/DPX LUVs at micro molar concentrations (Table 3). However, the N-terminal derived peptide RN6(1–45) was able to produce the same effect at 2.5-fold more concentration than its parental protein.

**Figure 2:**
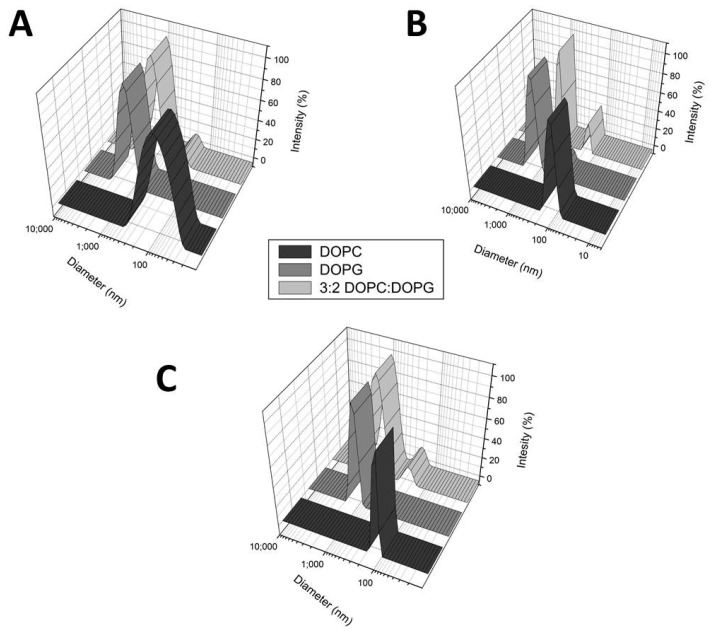
Liposome agglutination activity assayed by DLS. Plots show diameter size (nm) *versus* intensity of scattered light for DOPG, DOPC or DOPC:DOPG (3:2) in the presence of: (**A**) RNase3; (**B**) RNase6; and (**C**) RN6(1–45). Protein/peptide were added at 5 µM and mean diameter size of liposome population was registered after 15 min.

Additionally, dynamic light scattering (DLS) allowed us to investigate the physical changes of the LUV population upon interaction with RNase3, RNase6 and RN6(1–45). LUVs of DOPC, DOPG and mixture of DOPC/DOPG, with a vesicle diameter size of 100 nm, were prepared. RNase3, RNase6 and RN6(1–45) promoted the agglutination of charged LUVs in a short time course of 15 min (Figure 2). However, RNase6 and its N-terminal derived peptide were not able to agglutinate neutral LUVs, as previously observed by RNase3 [39].

### 2.2. Bactericidal Activity

The promising preliminary results on model membranes encouraged us to further investigate the protein and peptide mechanism of action at the bacterial cell level. Based on our previous characterization work on the antimicrobial activity of RNase3 [38], we analyzed here the human RNase6 and its derived N-terminal peptide cytotoxic mechanism on Gram-negative and Gram-positive bacteria.

To assess the antimicrobial activity of the human RNase3, RNase6 and its derived N-terminal peptide RN6(1–45) we determined their minimal bactericidal concentration (MBC) against three representative Gram-negative and Gram-positive species (Table 4).Complementarily, assessment of the protein and peptide antimicrobial activities was also performed by evaluating the reduction of bacterial cell viability using the *BacTiter-Glo* kit assay, which estimates the number of viable cells by quantification of ATP levels (Table S1). Additionally, RNase3, RNase6 and its derived N-terminal peptide RN6(1–45) were able to totally inhibit bacterial growth at low micro molar concentration (Table S2). Both human RNases displayed a high antimicrobial activity in a sub micro molar range against all tested Gram-positive and Gram-negative species. Remarkably, the N-terminal derived peptide RN6(1–45) was able to perform the same cytotoxic effect than its parental protein.

To characterize the cell selectivity of both human RNases and the N-terminal derived peptide, their hemolytic activity was tested on sheep RBCs, the concentration required to cause 50% hemolysis is reported as HC_50_ (Table 4). The HC_50_ values obtained for RNase3, RNase6 and RN6(1–45) showed that no hemolytic activity is present under the maximum concentration tested (20 µM), being at least 20- to 100-fold higher that the determined MBC values.

Further investigations on the bactericidal properties of the human RNase6 and its derived peptide were compared by assaying the membrane depolarization activity against two bacterial model species (*E. coli* and *S. aureus*). As previously described [37], RNase3 is able to interact with the Gram-negative and Gram-positive bacterial envelope, and can perturb the cell cytoplasmic membrane, producing half of the total membrane depolarization at concentrations below 1 µM (Table 5). It is worth noticing that comparable results were recorded for RNase6. In contrast, the antimicrobial peptide RN6(1–45) showed a slight decrement for its ability to depolarize bacterial membranes when compared to its parental protein.

In order to analyze the bactericidal kinetics of the two RNases and the RNase6 derived peptide we used the Live/Dead bacterial viability kit. The bacterial population viability was followed by the differential fluorescent staining of Syto 9 and propidium iodide (PI). Syto 9 can cross intact cell membranes, whereas PI stains damaged membrane dead cells. Therefore, the bacterial killing process was monitored as function of time (Table 6). Human RNase6 and its derived N-terminal peptide RN6(1–45) displayed similar bactericidal kinetics producing half of its total cytotoxic effect after several minutes of incubation. Comparable results were also obtained for RNase3.

**Figure 3:**
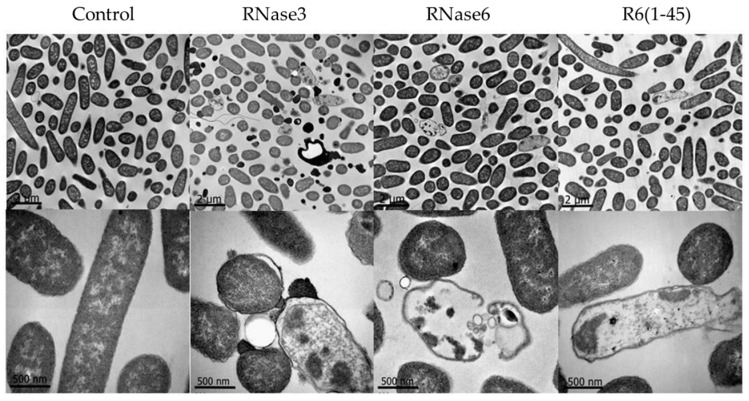
Transmission electron microscopy (TEM) micrographs for *E. coli* cultures incubated in the absence and presence of RNase3, RNase6 and RN6(1–45). Two magnifications (**upper** and **lower** panels) are shown for each condition to visualize the extent of bacteria aggregates and cell morphology. Scale bars correspond to 2 µm and 500 nm, respectively.

Additionally, further inspection of the bactericidal action at the Gram-positive and -negative cell envelope, was applied by electron microscopy. *E. coli* and *S. aureus* were examined by transmission electron microscopy (TEM) after 4 h of incubation with 5 µM of RNase3, RNase6 and the RN6(1–45) peptide (Figure 3). In accordance with the results presented above, both antimicrobial RNases and the N-terminal peptide showed a potent bactericidal effect against both *E. coli* and *S. aureus.* Both Gram-positive and -negative cells presented a complete disruption of the cell integrity, bacterial swelling, intracellular material spillage, bacterial cell wall layer detachment, and alteration of the cell morphology.

A distinctive feature of the RNase3 mechanism of action is the ability to promote bacterial agglutination of Gram-negative bacteria cells [41,42]. In order to assess whether RNase6 and its derived N-terminal peptide also shared this particular property, their minimal agglutination concentration (MAC) were determined (Table 7). Strikingly, the MAC values obtained showed a potent agglutinating activity for RNase6, which presented the same value as RNase3. Importantly, the N-terminal peptide RN6(1–45) retained significant agglutinating activity, being also able to promote bacterial agglutination at a micro molar range.

**Figure 4:**
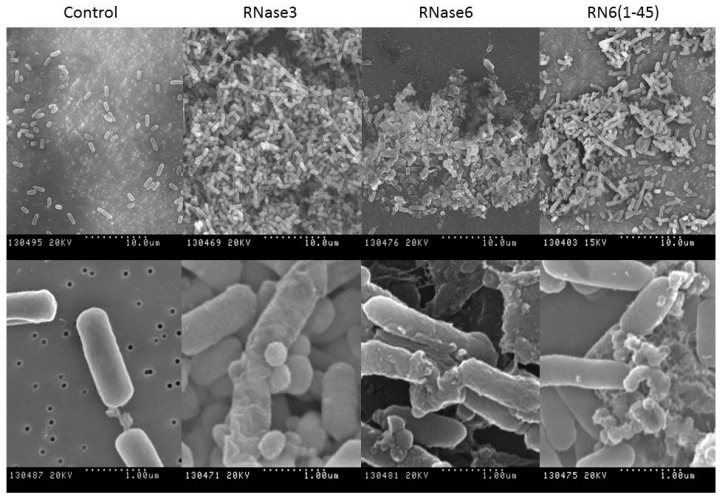
SEM micrographs for *E. coli* cultures incubated in the absence and presence of RNase3, RNase6 and RN6(1–45). Two magnifications (**upper** and **lower** panels) are shown for each condition to visualize the extent of bacteria aggregates and cell morphology. The magnification scale is indicated at the bottom of each micrograph.Finally, the agglutinating activity of the antimicrobial RNase6 and its derived N-terminal peptide was quantified by FACS (Figure 5 and Table 7). Comparable results were obtained by both RNase3 and 6, which, after 4h incubation, were able to induce the agglutination of most of the bacterial population. Interestingly, the antimicrobial peptide RN6(1–45) displayed a high agglutinating activity promoting a bacterial agglutination activity similar to its parental protein.

Henceforth, scanning electron microscopy (SEM) was applied in order to visualize cell population behavior and damage. SEM micrographs revealed tight densely populated bacterial aggregates after incubation with both antimicrobial RNases and the N-terminal derived peptide (Figure 4). In addition, cells were conspicuously damaged displaying a prominent loss of membrane integrity showing frequent blebs and loss of the baton-shaped cell morphology.

### 2.3. Mutant Design and Characterization

A closer look of the N-terminal region of RNase6 with the prediction software *Aggrescan3D* showed that RNase6 presented an aggregation prone region at the first 16 residues, as reported for RNase3 [3,40]. In order to confirm the presence of the spotted aggregation prone patch, we generated two RNase6 mutants targeting two key residues at the identified region: Trp1 and Ile13 (Figure 1 and Figure S1). In fact, mutation of residue 13 in both RNases sequences reduced the protein aggregation A3D score value, defined as a global indicator of the aggregation propensity/solubility of a protein structure. Interestingly, when we analyzed the A3D aggregation profiles we observed that while the I13A mutation in RNase3 caused a slight reduction (≈20%) of the value, the same mutation in the case of RNase6 abolished completely the aggregation propensity score (Figure S1). Structural comparison of the aggregation regions corroborated that mutation of Ile13 decreased the aggregative capacity of both proteins, being much more pronounced for RNase6 (Figure S1). On the other hand, the Trp1 is fully exposed at the protein surface and may perform an equivalent role to Trp35 in RNase3. Indeed, RNase 3-W35A mutant was found defective in its membrane interaction, lipid vesicle lysis, agglutination and bactericidal activities [38,43,44,45]. Additionally, an active site mutant (H15A) was used as a control reference, where the substitution of the His15 catalytic residue drastically impaired the protein enzymatic activity [34].

Results confirmed the involvement of both Trp1 and Ile13 residues in RNase6 antimicrobial action. In particular, both residues were critical for the protein cell agglutination activity (Table 8). Besides, the results also demonstrated that the hydrophobic patch at the N-terminal region of the protein is also related to the interaction with bacterial cell wall components. LPS binding assays for the two RNase6 mutants showed a decrease in their interaction affinities (Table 8). Moreover, both mutants displayed a poor antimicrobial activity against *E. coli* and *S. aureus* (Table 8). On the other hand, the tested active site mutant (H15A) retained its antimicrobial activity for both studied Gram-negative and Gram-positive species (Table S3).

## 3. Discussion

Antimicrobial RNases are small cationic proteins that belong to the vertebrate-specific RNaseA superfamily [46]. In this study, we have thoroughly characterized the antimicrobial mechanism of action of the human RNase6 and compared it along with the most studied human antimicrobial RNase, RNase3 [12,21]. The present results highlight that RNase6 also displays a high antimicrobial activity showing MBC values at sub micro molar for all tested Gram-positive and -negative bacterial species (Table 4). Kinetic viability assays showed that the antibacterial activity occurs in a matter of few minutes incubation time (Table 6). Furthermore, the results obtained by the fluorimetric DiSC3(5) assay showed that RNase6 displays a high membrane depolarization activity (Table 5) indicating that one of the main bactericidal route for these proteins takes place at the membrane level, as previously reported for RNase3. Applying rational mutation at the RNase scaffold and peptide synthesis approaches we have previously unveiled the main structural determinants for the antimicrobial action of human RNase3 [37,38,41,44,45,47,48]. Specifically, we demonstrated that the entirety of the RNase3 protein was not required for the antimicrobial action [38,44]. By applying prediction software for protein antimicrobial regions (*AMPA*) and by experimental proteolysis mapping, we located the key RNase3 antimicrobial region at the N-terminus [47,49]. Interestingly, a screening of RNase7 fragments also confirmed that the C-terminus was not able to reproduce the protein properties [27]. In fact, recent comparative results suggested that evolution has selected the N-terminal region of the vertebrate ribonucleases to encode the required structural determinants for antimicrobial action [3]. In the present study, we have characterized the N-terminal region of RNase6. The corresponding RN6(1–45) peptide displays almost the same antimicrobial activity than the whole protein; showing a fast and high bactericidal effect mediated by the destabilization of the bacterial membranes. On the other hand, the RNase6 also showed very low cytotoxicity levels against mammalian cells (Table 4). The results suggest that evolution has promoted the antimicrobial properties of RNases with a high selectivity towards pathogen cells.

Moreover, both RNase3 and 6 present the main common features of cationic antimicrobial peptides, presenting a positive net charge that would enable them to interact with the negatively charged bacterial cell envelopes, together with a high percentage of hydrophobic residues that could mediate the interaction with the membrane lipid bilayer [7]. Previous structural characterization of RNase3 confirmed its mechanism of action in a membrane model system [39,43]. In tune with these facts, internal fluorescence tryptophan spectra of the RNase6 showed how the protein is able to interact with negatively charged membranes, as visualized by the blue-shift of the fluorescent emission wavelength (Table 1). Additionally, the structural determinants required for membrane interaction were retained by the N-terminal region, as a significant blue-shift was also registered for the RNase6 derived peptide. On the other hand, the results indicated that RNase6 is able to interact with the negatively charged LPS molecules of the Gram-negative surface (Table 2), serving as the first point of anchor to exert the membranolytic activity. Also, the ANTS/DPX fluorescence assay showed that RNase6 is able to destabilize lipid bilayers very efficiently, presenting a high membrane lysis at low micro molar concentrations (Table 3). Our previous work identified a key antimicrobial region for RNase3 (residues 24 to 45) essential for membrane leakage, depolarization and LPS binding [47]. Moreover, recent comparative analysis of human RNases N-terminus peptides confirmed their structuration, adopting a secondary helical conformation, in the presence of sodium dodecyl sulfate (SDS) and LPS micelles [3]. Interestingly, we have proven here that the N-terminal region of the homologue RNase6 is also able to reproduce the membrane destabilization properties of the whole protein. Nonetheless, significant differences between the two proteins were observed regarding the LPS binding activity; where the RNase6 N-terminal region shows reduced LPS binding. Differences at the predicted protein LPS binding residues may account for this data [3,42].

**Figure 5:**
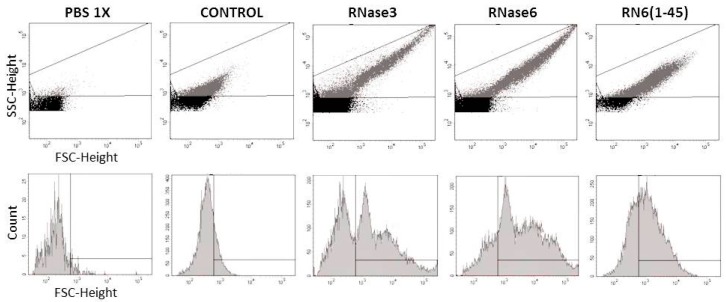
Bacterial agglutination measured by FACS. *E. coli* cultures were incubated in the absence and presence of RNase3, RNase6 and RN6(1–45) at a final concentration of 5 μM for 4 h. Low-angle forward scattering (FSC-H) is represented on the *x*-axis and the side scattering (SSC-H) on the *y*-axis to analyze the size and complexity of the cell cultures. Plots show density of cell population distribution. Buffer background is shown in black and cell population in grey.

Another important feature for RNase3 antimicrobial activity is the ability to agglutinate Gram-negative bacterial cells in a LPS binding dependent manner [42]. We previously demonstrated that RNase3 upon interaction with the negatively charged surfaces of the bacteria underwent conformational changes that triggered the amyloid-like self-aggregation of the protein ensuing in bacterial agglutination and eventual cell death [19,40]. Antimicrobial peptides endowed with a cell agglutinating activity would prevent dissemination of the infectious focus and facilitate the infection clearance by the host innate cells [19,50]. Interestingly, RNase6 is also able to aggregate both lipid vesicles and Gram-negative bacteria in a micro molar range. Electron micrographs not only showed the evident cell damage that RNase6 antimicrobial action produces but they also revealed densely packed bacterial aggregates, as observed for RNase3 [37]. Quantification of the agglutinating activity of the RNase6 by FACS showed that the totality of the bacterial population is agglutinated at 5 µM protein concentration (Figure 5). Again, the N-terminal region of RNase6 retained the agglutinating properties of the whole protein (Table 7). To confirm this hypothesis, two point mutants were designed at the spotted aggregation propensity region (W1A and I13A). The results confirmed that substitution of both hydrophobic residues reduced considerably the bacterial agglutination activity and antimicrobial action (Table 8). Significant reduction of the protein LPS interaction was also obtained for both mutant variants (Table 8). Interestingly, Trp1 is unique to RNase6 lineage and is conserved in all the sequenced Old World primate genes [51,52,53]. On the other hand, previous studies from our laboratory highlighted the involvement of Ile13 in the RNase3 agglutination properties [40,45]. Moreover, the residue is present in the three main antimicrobial RNases within the family (Figure 1). Comparison of the 8 N-terminal peptides from the human canonical RNases confirmed the direct correlation between the hydrophobic patch and the protein agglutination and bactericidal properties [3]. Besides, no reduction of the RNase6 antimicrobial activity was observed for the H15A active site mutant (Table S3).Therefore, our present results are confirming that residues Trp1 and Ile13 play a crucial role in RNase6 bacterial cell surface interaction, membrane disruption and bacterial agglutination.

## 4. Materials and Methods

### 4.1. Materials and Strains

DOPC and DOPG were from Avanti Polar Lipids (Alabaster, AL, USA). ANTS, DPX and BC were purchased from Invitrogen (Carlsbad, CA, USA). LPS from *E. coli* serotype 0111:B4 were purchased from Sigma-Aldrich (St. Louis, MO, USA). PD-10 desalting columns with Sephadex G-25 were from GE Healthcare (Waukesha, WI, USA). RNase6(1–45) peptide was purchased from Genecust (Dudelange, Luxembourg). Strains used were *Escherichia coli* (BL21; Novagen, Madison, WI, USA), *Staphylococcus aureus* (ATCC 502A; Manassas, VA, USA), *Acinetobacter baumannii* (ATCC 15308; Manassas, VA, USA), *Pseudomonas aeruginosa* (ATCC 47085; Manassas, VA, USA), *Micrococcus luteus* (ATCC 7468; Manassas, VA, USA) and *Enterococcus faecium* (ATCC 19434; Manassas, VA, USA).

### 4.2. Protein Expression and Purification

Wild-type RNase3 was obtained from a synthetic gene [54]. Human RNase6 was obtained from DNA 2.0 (Menlo Park, CA, USA). Both genes were subsequently cloned into pET11c vectors. Mutations into the *RNase6* gene were introduced using the Quick change™ site-directed mutagenesis kit (Santa Clara, CA, USA) following the manufacturers procedure. *E. coli* BL21(DE3) (Novagen, Madison, WI, USA) competent cells were transformed with the pET11c/RNase6 and RNase3 plasmids. The expression protocol was optimized in Terrific broth (TB). For high yield expression, bacteria were grown in TB, containing 400 µg/mL ampicillin. Recombinant RNase6 was expressed in *E. coli* BL21(DE3) (Novagen, Madison, WI, USA) cells after induction with 1 mM IPTG (St. Louis, MO, USA), added when the culture showed an OD_600_ of 0.6. The cell pellet was collected after 4 h of culture at 37 °C. Cells were resuspended in 10 mM Tris-HCl, 2 mM EDTA, pH 8, and sonicated at 50 watts for 10 min with 30-s cycles. After centrifugation at 15,000× *g* for 30 min, the pellet fraction containing inclusion bodies was processed as follows: the pellet fraction was washed with 50 mM Tris-HCl, 2 mM EDTA, 0.3 M NaCl, pH 8, and after centrifugation at 20,000× *g* for 30 min, the pellet was dissolved in 12 mL of 6 M guanidine HCl, 0.1 M Tris-acetate, 2 mM EDTA, pH 8.5, containing 80 mM GSH (St. Louis, MO, USA), and incubated under nitrogen for 2 h at room temperature. The protein was then refolded by a rapid 100-fold dilution into 0.1 M Tris-HCl, pH 7.5, containing 0.5 M l-arginine, and GSSG (St. Louis, MO, USA) was added to obtain a GSH/GSSG ratio of 4. Dilution in the refolding buffer was adjusted to obtain a final protein concentration of 30–150 µg/mL. The protein was incubated in refolding buffer for 48–72 h at 4 °C. The folded protein was then concentrated, dialyzed against 0.015 M Tris-HCl, pH 7, and purified by cation exchange chromatography on a Resource S column equilibrated with the same buffer. ECP was eluted with a linear NaCl gradient from 0 to 2 M in 0.015 M Tris-HCl, pH 7 buffer. Further purification was achieved by a second reverse phase chromatography on a Vydac C4 column (Grace-Alltech, Bannockburn, IL, USA). The homogeneity of the purified proteins was checked by 15% SDS-PAGE and Coomassie Blue staining and by N-terminal sequencing.

### 4.3. Minimal Bactericidal Concentration (MBC)

Antimicrobial activity was expressed as the MBC_100_, defined as the lowest protein concentration that completely kills a microbial population. The MBC of each protein/peptide was determined from two independent experiments performed in triplicate for each concentration. Bacteria cells were incubated at 37 °C overnight in LB broth and diluted to give approximately 5 × 10^5^ CFU (colony forming units)/mL. The bacterial suspension was incubated in LB with peptides at various concentrations (0.1–20 μM) at 37 °C for 4 h. Samples were plated on to Petri dishes and incubated at 37 °C overnight.

### 4.4. Bacterial Viability Assays

Kinetics of bacterial survival were determined using the Live/Dead bacterial viability kit (Molecular Probes, Invitrogen) in accordance with the manufacturer’s instructions. Bacterial strains were grown at 37 °C to an optical density (OD_600_) of 0.2, centrifuged at 5000× *g* for 5 min, and stained in a 0.85% NaCl solution. Fluorescence intensity was continuously measured after protein or peptide addition (10 µM) using a Cary Eclipse spectrofluorimeter (Varian Inc., Palo Alto, CA, USA). To calculate bacterial viability, the signal in the range of 510 to 540 nm was integrated to obtain the Syto 9 signal (live bacteria) and that in the range of 620 to 650 nm was integrated to obtain the propidium iodide (PI) signal (dead bacteria). The percentage of live bacteria was represented as a function of time, and t_50_ values were calculated by fitting the data to a simple exponential decay function with Origin 7.0 (OriginLab Corporation; Northampton, MA, USA).

Alternatively, bacterial viability was assayed using the *BacTiter-Glo* microbial cell viability kit (Promega; Fitchburg, WI, USA) that estimates the number of viable cells by ATP quantification using a fluorescence assay. Briefly, proteins and peptides were dissolved in 10 mM sodium phosphate buffer, 0.1 M NaCl (pH 7.4), serially diluted from 20 to 0.1 µM, and tested against the bacterial species (OD_600_ ~ 0.2) for 4 h of incubation time. Fifty microliters of culture were mixed with 50 µL of *BacTiter-Glo* reagent in a microtiter plate according to the manufacturer’s instructions and incubated at room temperature for 15 min. Luminescence were read on a Victor3 plate reader (Perkin-Elmer, Waltham, MA, USA) with a 3-s integration time. Fifty percent effective dose concentrations (ED_50_) were calculated by fitting the data to a dose–response curve with Origin 7.0.

### 4.5. Bacterial Cell Membrane Depolarization Assay

Membrane depolarization was performed using the membrane potential-sensitive DiSC3(5) fluorescent probe as described previously [41,55]. After interaction with intact cytoplasmic membrane, the fluorescent probe DiSC3(5) was quenched. After incubation with the antimicrobial protein or peptide, the membrane depolarization was induced the probe was released to the medium, ensuing in an increase of fluorescence that can be quantified and monitored as a function of time. Bacterial cultures were grown at 37 °C to an OD_600_ of 0.2, centrifuged at 5.000× *g* for 7 min, washed with 5 mM HEPES-KOH, 20 mM glucose (pH 7.2), and resuspended in 5 mM HEPES-KOH 20 mM glucose 100 mM KCl (pH 7.2) to an OD_600_ of 0.05. DiSC3(5) was added to a final concentration of 0.4 µM, and changes in the fluorescence were continuously recorded after the addition of protein (from 0.01 to 20 µM) in a Victor3 plate reader. Effective dose values (ED_50_) were estimated from nonlinear regression analysis.

### 4.6. Minimal Agglutination Activity (MAC)

Bacterial cells were grown at 37 °C to an OD_600_ of 0.2, centrifuged at 5000× *g* for 2 min. One hundred microliters of the bacterial suspension was treated with increasing protein or peptide concentrations (from 0.01 to 20 µM) and incubated at 37 °C for 1 h. The aggregation behavior was observed by visual inspection, and the agglutinating activity is expressed as the minimum agglutinating concentration of the sample tested, as previously described [42].

### 4.7. Fluorescence Activated Cell-Sorting (FACS)

Bacterial cells were grown at 37 °C to mid-exponential phase (OD_600_ of 0.6), centrifuged at 5000× *g* for 2 min, resuspended in 10 mM sodium phosphate buffer and 100 mM NaCl (pH 7.4) to give a final OD_600_ of 0.2 and pre-incubated for 20 min. A 500-µL aliquot of the bacterial suspension was incubated with 5 µM protein/peptide for 4 h. After incubation, 25,000 cells were subjected to FACS analysis using a FACS Calibur cytometer (BD Biosciences; Franklin Lakes, NJ, USA) and a dot-plot was generated by representing the low-angle forward scattering (FSC-H) in the *x*-axis and the side scattering (SSC-H) in the *y*-axis to analyze the size and complexity of the cell cultures.

### 4.8. Scanning Electron Microscopy (SEM)

Scanning electron microscopy (SEM) samples were prepared as previously described [56]. Bacterial culture of *S. aureus* and *E. coli* were grown at 37 °C to mid-exponential phase (OD_600_ of 0.2) and incubated with proteins or peptide (5 μM) at 37 °C. Sample aliquots (500 μL) were taken after up to 4 h of incubation and prepared for SEM analysis as previously described [41]. The micrographs were viewed at a 15-kV accelerating voltage on a Hitachi S-570 scanning electron microscope (Hitachi, Ltd.; Chiyoda, Tokio, Japan), and a secondary electron image of the cells for topography contrast was collected at several magnifications.

### 4.9. Transmission Electron Microscopy

Transmission electron microscopy (TEM) samples were prepared as previously described [56]. *E. coli* and *S. aureus* cultures were grown to an OD_600_ of 0.2 and incubated at 37 °C with 5 µM proteins or peptides for 4 h. After treatment, bacterial pellets were prefixed with 2.5% glutaraldehyde and 2% paraformaldehyde in 0.1 M cacodylate buffer at pH 7.4 for 2 h at 4 °C and postfixed in 1% osmium tetroxide buffered in 0.1 M cacodylate at pH 7.4 for 2 h at 4 °C. The samples were dehydrated in acetone (50%, 70%, 90%, 95%, and 100%). The cells were immersed in Epon resin, and ultrathin sections were examined in a JEOL JEM 2011 instrument (JEOL, Ltd., Tokyo, Japan).

### 4.10. Hemolytic Activity

Fresh sheep RBCs (red blood cells) (Oxoid Inc.; Nepean, ON, Canada) were washed three times with PBS (35 mM phosphate buffer and 0.15 M NaCl (pH 7.4)) by centrifugation for 5 min at 3000× *g* and resuspended in PBS at 2 × 10^7^ cells/mL. RBCs were incubated with protein/peptide at 37 °C for 4 h and centrifuged at 13,000× *g* for 5 min. The supernatant was separated from the pellet and its absorbance measured at 570 nm. The 100% hemolysis was defined as the absorbance obtained by sonicating RBCs for 10 s. HC_50_ was calculated by fitting the data to a sigmoidal function with Origin 7.0.

### 4.11. Liposome Preparation

Large Unilamellar Vesicles (LUVs) containing DOPC, DOPG or DOPC/DOPG (3:2 molar ratio) of a defined size were obtained from a vacuum drying lipid chloroform solution by extrusion through 100 nm polycarbonate membranes. The lipid suspension was frozen and thawed ten times before extrusion. A 1 mM stock solution of liposome suspension was prepared in 10 mM sodium phosphate, 100 mM NaCl, pH 7.4.

### 4.12. Intrinsic Tryptophan Fluorescence Analysis

Tryptophan fluorescence emission spectra were recorded using a 280 nm excitation wavelength. Slits were set at 2 nm for excitation and 5–10 nm for emission. Emission spectra were recorded from 300–400 nm at a scan rate of 60 nm/min in a 10 mm × 10 mm cuvette, with stirring immediately after sample mixing. Protein/peptide spectra at 0.5 μM in 10 mM Hepes buffer, pH 7.4, were obtained at 37 °C in the absence or presence of 200 μM liposome suspension or 100 μM LPS micelles. Fluorescence measurements were performed on a Cary Eclipse spectrofluorimeter (Agilent Technologies, Bath, UK). Spectra in the presence of liposomes or LPS were corrected for light scattering by subtracting the corresponding background. For each condition three spectra was averaged. The maximum of the fluorescence spectra was calculated fitting the data to a log-normal distribution function with Origin 7.0.

### 4.13. Liposome Leakage

The ANTS/DPX liposome leakage fluorescence assay was performed as previously described [43]. Briefly, a unique population of LUVs DOPC/DOPG (3:2) was prepared to encapsulate a solution containing 12.5 mM ANTS, 45 mM DPX, 20 mM NaCl, and 10 mM Tris/HCl, pH 7.5. The ANTS/DPX liposome stock suspension was diluted to 30 μM and incubated at 37 °C with protein/peptide, serially diluted from 20 to 0.1 μM in a microtiter plate. Fluorescence measurements were performed on a Victor3 plate reader (PerkinElmer, Waltham, MA, USA). ED_50_ values were calculated by fitting the data to a dose–response curve with Origin 7.0.

### 4.14. Liposome Aggregation

Liposome aggregation was assessed by Dynamic Light Scattering (DLS). A unique population of LUVs DOPC, DOPG or DOPC/DOPG was incubated at 37 °C with 5 μM of each protein/peptide for 15 min. Particle size distribution was measured with a Zetasizer Nano ZS (Malvern Instruments Ltd., Worcestershire, UK). Polydispersity of LUV population was also analyzed. Size radius was plotted *versus* protein concentration.

### 4.15. LPS Binding Fluorimetric Assay

Protein binding to LPS was assessed using the fluorescent probe BODIPY^®^ cadaverine (BC) (St. Louis, MO, USA). BC binds strongly to native LPSs, specifically recognizing the lipid A portion. When a protein that interacts with LPSs is added, the BC is displaced from the complex, and its fluorescence is increased. LPS-binding assays were carried out in 10 mM Hepes buffer at pH 7.2. The displacement assay was performed by a microtiter plate containing a stirred mixture of either LPS (10 μg/mL) and BC (10 μM). Proteins and peptide were serially diluted from 20 to 0.1 μM. Fluorescence measurements were performed on a Victor3 plate reader (PerkinElmer, Waltham, MA, USA).

### 4.16. Aggrescan3D Analysis

Aggregation propensity of RNase6 was calculated by *Aggrescan*3D server [57]. The A3D value was calculated for each protein residue as described. The analysis was performed with the dynamic mode enabled and the distance of aggregation analysis was defined to 10 Å.

### 4.17. Minimal Inhibitory Concentration (MIC)

Minimal inhibitory concentration expressed as MIC_100_, defined as the lowest protein concentration that completely inhibits microbial growth. The MIC of each protein/peptide was determined from two independent experiments performed in duplicate for each concentration. Bacteria cells were incubated at 37 °C overnight in LB broth and diluted to give approximately 5 × 10^5^ CFU (colony forming units)/mL. The bacterial suspension was incubated in LB with peptides at serially diluted concentrations (0.1–20 μM) at 37 °C for 20 h. Bacterial growth inhibition was determined by measuring the optical density (OD) of the samples at a wavelength of 570 nm.

## 5. Conclusions

In this work, we have characterized for the first time the bactericidal properties of the human RNase6. Our data suggest that the antimicrobial mechanism of action encompasses two steps: the protein firstly interacts with the negatively charged envelopes of the bacterial cell, promptly followed by a combination of membrane destabilization and bacterial agglutination. Furthermore, the N-terminal domain of the protein was proven to retain the antimicrobial properties of the whole protein; encouraging us to use the scaffold of the human RNase6 for the future development of new peptide based antimicrobial agents.

## Figures and Tables

**Table 1:** Tryptophan fluorescence in the presence of lipid vesicles and LPS micelles for RNase3, RNase6 and RN6(1–45).

Protein/Peptide	λmax for Fluorescence Emission (nm)				
	Buffer	DOPC ^a^	DOPG ^a^	DOPC:DOPG(3:2) ^a^	LPS ^a^
RNase3	343	-	3	3	4
RNase6	345	-	2	6	4
RN6(1–45)	343	-	8	9	3

^a^ The shift in the maximum emission wavelength compared with the reference value for buffer sample is indicated.

**Table 2:** LPS-binding affinity of RNase3, RNase6 and RN6(1–45).

Protein/Peptide	LPS Binding	
	ED_50_ (µM) ^a^	Max (%) *
RNase3	2.54 ± 0.14	100
RNase6	2.83 ± 0.22	76
RN6(1–45)	5.31 ± 1.41	27

^a^ ED_50_ values are given as mean ± standard error of the mean (SEM); * 100% refers to a total displacement, whereas 0% corresponds to no displacement of the fluorescent dye, indicating no affinity for LPS.

**Table 3:** Liposome leakage activity of RNase3, RNase6 and RN6(1–45).

Protein/Peptide	Liposome Leakage (µM)	
	ED_50_ (µM) ^a^	Max (%) *
RNase3	0.7 ± 0.1	100
RNase6	1.5 ± 0.5	100
RN6(1–45)	4.0 ± 0.5	100

^a^ ED_50_ values are given as mean ± SEM; * 100% refers to the total leakage of liposome content. The ANTS/DPX liposome leakage fluorescence assay was performed using DOPC/DOPG vesicles as described in the methodology.

**Table 4:** Minimal bactericidal concentration (MBC_100_) and hemolytic activity (HC_50_) of RNase3, RNase6 and RN6(1–45).

Protein/Peptide	MBC_100_ (μm) ^a^						
	*E. coli*	*P. aeruginosa*	*A. baumanii*	*S. aureus*	*M. luteus*	*E. faecium*	HC_50_ (μm) *
RNase3	0.35 ± 0.02	0.20 ± 0.01	0.40 ± 0.03	0.40 ± 0.03	0.65 ± 0.08	0.90 ± 0.14	>20
RNase6	0.90 ± 0.14	0.90 ± 0.14	0.65 ± 0.08	1.87 ± 0.56	1.35 ± 0.23	1.35 ± 0.23	>20
RN6(1–45)	1.35 ± 0.23	0.40 ± 0.03	0.65 ± 0.08	1.87 ± 0.56	1.35 ± 0.23	1.35 ± 0.23	>20

^a^ The MBC_100_ was calculated as described in Materials and Methods by colony forming units (CFU) counting on plated Petri dishes. All values are averaged from three replicates of two independent experiments. Values are given as mean ± SEM; * Hemolytic activity was assayed on sheep red blood cells.

**Table 5:** Depolarization activity on *E.coli* and *S.aureus* cells determined for RNase3, RNase6 and RN6(1–45).

Protein/Peptide	Depolarization (µM)			
	*E. coli*		*S. aureus*	
	ED_50_	Depol_max_ *	ED_50_	Depol_max_ *
RNase3	0.5 ± 0.1	100.5 ± 3.8	0.7 ± 0.2	100.5 ± 6.7
RNase6	0.6 ± 0.1	64.1 ± 4.2	0.9 ± 0.1	71.7 ± 1.5
RN6(1–45)	1.1 ± 0.3	69.7 ± 5.3	1.2 ± 0.2	76.2 ± 5.6

* Maximum fluorescence value reached at the final incubation time with 5 µM of the proteins and peptides. Membrane depolarization was performed using the membrane potential-sensitive DiSC3(5) fluorescent probe as described in the Methodology. Values are given as mean ± SEM.

**Table 6:** Bactericidal kinetics on *E. coli* and *S. aureus* cells determined by the Live/Dead assay for RNase3, RNase6 and RN6(1–45).

Protein/Peptide	Bacterial Viability Assay			
	*E. coli*		*S. aureus*	
	t_50_ (min) *	Viability (%) *	t_50_ (min) *	Viability (%) *
RNase3	10.6 ± 0.1	4.2 ± 0.3	7.1 ± 0.1	9.3 ± 0.2
RNase6	15.4 ± 0.1	8.8 ± 0.1	18.0 ± 0.4	12.9 ± 0.8
RN6(1–45)	13.1 ± 0.1	5.2 ± 0.1	5.5 ± 0.1	5.8 ± 0.2

* Viability percentage and half time were determined with the Live/Dead kit after 4 h of incubation of mid-log-phase-grown *E. coli* and *S. aureus* cultures with 5 μM of protein and peptides. The percentage of viability (%) and half time of viability (t_50_) after incubation with the proteins are shown. The percentage of live bacteria was represented as a function of time, and t_50_ values were calculated by fitting the data to a simple exponential decay function with Origin 7.0. Values are given as mean ± SEM.

**Table 7:** MAC and bacterial agglutination percentage of RNase3, RNase6 and RN6(1–45) for *E. coli* cell cultures.

Protein/Peptide	MAC (µM) ^a^	Agglutination Activity (%) *
RNase3	0.20 ± 0.05	60.35 ± 0.50
RNase6	0.20 ± 0.05	80.64 ± 0.50
RN6(1–45)	5 ± 0.50	66.22 ± 0.50

^a^ *E. coli* cells were treated with increasing protein or peptide concentrations (from 0.01 to 20 μM); * Agglutination activity percentage registered by incubation of bacteria culture with 5 μM protein concentration for 4 h were calculated by Fluorescence-Activated Cell Sorting (FACS) as described in the Methodology. Values are given as mean ± SEM.

**Table 8:** MBC, MAC and LPS Binding activities of RNase3, RNase6 and RNase6 mutants.

Protein/Peptide	MBC_100_(µM) ^a^		MAC (µM)		LPS Binding Assay	
	*E. coli*	*S. aureus*	*E. coli*	*S. aureus*	ED_50_ (µM)	Max (%) *
RNase3	0.35 ± 0.01	0.40 ± 0.10	0.22 ± 0.01	>5	2.54 ± 0.16	99.89 ± 4.20
RNase6	0.90 ± 0.14	1.87 ± 0.56	0.22 ± 0.01	>5	2.63 ± 0.23	75.89 ± 4.41
RNase6-W1A	1.87 ± 0.56	3.75 ± 0.78	>5	>5	3.37 ± 0.34	38.55 ± 2.32
RNase6-I13A	3.75 ± 0.78	>5	>5	>5	4.60 ± 0.43	27.43 ± 1.22

^a^ The MBC_100_ was calculated as described in Materials and Methods. MBC_100_ values were calculated by CFU counting on plated Petri dishes. All values are averaged from three replicates of two independent experiments; * 100% refers to a total displacement, whereas 0% stands for no displacement of the dye, indicating no binding. Values are given as mean ± SEM.

## Supplementary Materials

Supplementary materials can be found at http://www.mdpi.com/1422-0067/17/4/552/s1.

## Abbreviations

ANTS	8-aminonaphthalene-1,3,6-trisulfonic acid
BC	BODIPY^®^ cadaverine
CFU	colony forming units
DLS	dynamic light scattering
DOPC	1,2-dioleoyl-sn-glycero-3-phosphocholine
DOPG	1,2-dioleoyl-sn-glycero-3-phosphoglycerol
DPX	*p*-xylenebispyridinium bromide
ED_50_	50% effective dose
FACS	fluorescent activated cell-sorting
HC	hemolytic activity
LB	Luria-Bertani media
LPS	lipopolysaccharide
LUV	large unilamellar vesicle
MAC	minimal agglutination concentration
MIC	minimal inhibitory concentration
PBS	phosphate buffer saline
RBC	red blood cell
RNase	Ribonuclease
SDS	sodium dodecyl sulfate
SEM	scanning electron microscopy
TB	terrific broth media
TEM	transmission electron microscopy
