# Supplementary material for: Insights into the Antimicrobial Mechanism of Action of Human RNase6: Structural Determinants for Bacterial Cell Agglutination and Membrane Permeation
Source: Int J Mol Sci. 2016 Apr 13;17(4):552. doi: 10.3390/ijms17040552 (PMC4849008; doi:10.3390/ijms17040552)
Supplement: Supplementary file 1 [file ijms-17-00552-s001.pdf]

Supplementary Materials: Insights into the Antimicrobial Mechanism of Action of Human RNase6: Structural Determinants for Bacterial Cell Agglutination and Membrane Permeation

David Pulido, Javier Arranz-Trullén, Guillem Prats-Ejarque, Diego Velázquez, Marc Torrent, Mohammed Moussaoui and Ester Boix

Table S1. Comparison of the proteins and peptide antimicrobial activities determined by the *BacTiter-Glo* bacterial viability assay.

Protein/Peptide	ED_{50} (μM) [a]	
	E. coli	*S aureus*
RNase3	0.4 ± 0.1	0.9 ± 0.2
RNase6	1.1 ± 0.2	1.8 ± 0.5
RN6(1–45)	2.1 ± 0.5	2.2 ± 0.1

[a] Fifty percent effective dose concentrations (ED_{50}) were calculated by fitting the data to a dose-response curve. Values are given as mean ± SEM.

Table S2. Minimal inhibitory concentration (MIC) of RNase3, RNase6 and RN6(1–45).

Protein/Peptide	MIC_{100} (μM) [a]					
	E. coli	*P. aeruginosa*	*A. baumanii*	*S. aureus*	*M. luteus*	*E. faecium*
RNase3	0.31	0.15	0.31	0.31	0.62	1.25
RNase6	0.62	0.62	0.62	1.25	1.25	1.25
RN6(1–45)	1.25	0.62	0.93	2.5	1.25	1.25

[a] The 100% Minimal inhibitory concentration (MIC_{100}) was calculated as described in Materials and Methods. All values are averaged from two replicates of two independent experiments.

Table S3. Comparison of RNase6 and RNase6-H15A mutant antimicrobial activity.

Protein/Peptide	MBC_{100} (μM) [a]	
	E. coli	*S aureus*
RNase6	0.92 ± 0.05	1.87 ± 0.56
RNase6-H15A	0.92 ± 0.14	1.87 ± 0.20

[a] The 100% Minimal bactericidal concentration (MBC_{100}) was calculated as described in Materials and Methods. MBC_{100} values were calculated by CFU counting on plated Petri dishes. All values are averaged from three replicates of two independent experiments. Values are given as mean ± SEM.

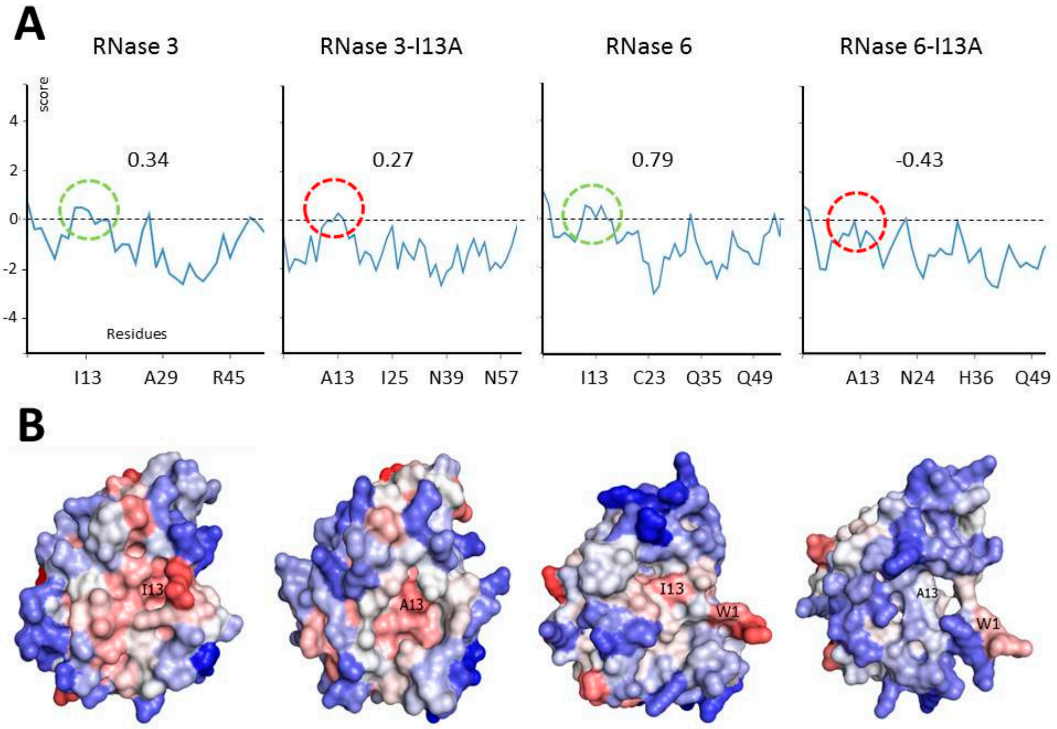

Figure S1. Aggregation propensity prediction ofRNase3 (**A**); RNase6 (**B**) and their I13A respective mutants. The 3D structure of RNase3 and RNase6 were built using the protein data bank (PDB) ID:4A2O [35] and PDB ID:4X09) [34] respectively. (**A**) The aggregation profile of the polypeptide chain is represented as a line plot using the calculated A3D score values. Residues with positive scores in the analyzed structure, therefore predicted as aggregation-prone, are indicated at the top profiles. A3D score values for residue 13 are depicted in each profile, green circles show the aggregation propensity for wild type proteins and red circles highlight the decrease of the aggregation propensity in mutants; (**B**) Protein structure is coloured according to the A3D score of the residues. Areas with high-predicted aggregation propensity are labelled using a **red** gradient and areas with high-predicted solubility are labelled with a **blue** gradient. **White** areas are not predicted to influence aggregation (A3D score ≈ 0).
